# Enhancing Named Entity Recognition for immunology and immune-mediated disorders

**DOI:** 10.3389/fimmu.2025.1613479

**Published:** 2026-02-04

**Authors:** Songyue Chen, Jinshan Che, Mingming Sun, Yuhong Wang

**Affiliations:** 1 Department of Rheumatology and Immunology, First Affiliated Hospital of Bengbu Medical University, Bengbu, China; 2 Fourth Clinical College of Xinxiang Medical College, Xinxiang Central Hospital, Xinxiang, China

**Keywords:** named entity recognition, biomedical NLP, immunology, structural span encoding, constraint-based decoding

## Abstract

**Introduction:**

Named Entity Recognition (NER) in the biomedical domain, particularly within immunology and immune-mediated disorders, presents unique challenges due to the presence of complex, nested, and overlapping entities. Existing NER systems often struggle with the specialized terminologies and contextual ambiguity of immunological texts, which limits their effectiveness in downstream biomedical applications.

**Methods:**

To address these challenges, we propose a domain-specific NERframework that integrates structured span encoding and knowledge-guided decoding. The framework is designed to enhance recognition accuracy under low-resource and weak supervision conditions by combining a hierarchical span encoder (SpanStructEncoder) with a constraint-based decoding strategy (Contextual Constraint Decoding, CCD). We evaluate our model on three immunology-specific datasets: the NCBI Disease Corpus (immune-related diseases), SNPPhenA (genetic variants and phenotype associations), and HLA-SPREAD (HLA-disease and drug-response relations). These datasets were selected because they represent key immunological concepts such as cytokines, immune cell types, and genetic markers that underlie immune responses and disease mechanisms.

**Results and discussion:**

Experimental results demonstrate that our model achieves consistent improvements in F1-score over strong biomedical baselines including BioGPT, BioLinkBERT, and SciFive. Our results confirm that incorporating structured span representations and ontology-aware decoding significantly improves entity extraction for immunology-related texts. The proposed framework provides a robust and interpretable solution for immunology-focused biomedical text mining, facilitating applications in literature curation, biomarker discovery, and clinical decision support.

## 1 Introduction

Named Entity Recognition (NER) is an essential task in the biomedical domain, especially in immunology and immune-mediated disorders, due to the rapid growth of scientific literature and clinical data. Identifying entities like immune cell types, cytokines, disease names, biomarkers, and therapeutic agents is crucial for knowledge extraction, literature mining, and clinical decision-making Li et al. (1). However, general-purpose NER models often fail to capture the specialized terms and relationships found in immunological texts, leading to incomplete or inaccurate annotations Sahin et al. (2). This gap highlights the need for NER systems tailored specifically to the immunology domain Jian et al. (3). These systems not only improve biomedical knowledge curation but also support the integration of multi-omics data and assist in the discovery of new insights into immune-mediated diseases Mi and Yi (4). Furthermore, as personalized medicine becomes more important in treating autoimmune diseases, allergies, and cancer immunotherapy, accurate entity extraction is crucial for creating patient profiles and enabling targeted treatments Weber et al. (5).

Early approaches to immunology-specific NER relied on structured linguistic patterns and terminological resources Hernandez-Lareda and Auccahuasi (6). These systems used curated dictionaries, synonym lists, and pattern-based rules to detect relevant biomedical terms in predefined contexts Khouya et al. (7). While these methods were precise and interpretable by domain experts, they required significant manual effort and struggled with new or unseen terms Bade et al. (8). Their inability to adapt to evolving language and capture nested structures limited their scalability in broader immunological datasets Yossy et al. (9). These limitations led to a shift toward more adaptive approaches Zhang et al. (10).

Later developments introduced probabilistic modeling techniques to increase flexibility and learning capacity in biomedical text processing Ushio and Camacho-Collados (11). Methods like Conditional Random Fields (CRF), Support Vector Machines (SVM), and Hidden Markov Models (HMM) were widely adopted, as they could learn patterns from annotated data and generalize to related contexts Ray et al. (12). These models captured dependencies among tokens and incorporated various syntactic and semantic features, improving recall and consistency across immunology subdomains Chen et al. (13). However, their reliance on high-quality labeled data and the need for extensive feature engineering limited their scalability Au et al. (14). As biomedical literature grew in volume and complexity, models capable of capturing deeper semantic relationships became essential Yu et al. (15).

Recent advancements have introduced deep learning architectures and pretrained models specifically fine-tuned for biomedical applications Li and Meng (16). Architectures like BiLSTM-CRF combinations enabled automatic feature learning from sequences, significantly reducing the need for handcrafted features. Models like BioBERT, SciBERT, and PubMedBERT—pretrained on vast biomedical corpora—have further improved performance by capturing domain-specific semantics and contextual nuances Zhang et al. (17). These models have proven effective in recognizing complex biomedical entities, but challenges remain in entity disambiguation, low-resource settings, and model generalization Taher et al. (18). Moreover, pretrained models may not always be well-suited to the terminological complexity of immunology, limiting their clinical utility in this specialized field.

To address the limitations of symbolic, machine learning, and general pretrained models, we propose a domain-adaptive NER framework specifically designed for immunology and immune-mediated disorders. Our approach integrates domain-specific knowledge with state-of-the-art language models, effectively leveraging both contextual and ontological information. By incorporating immunology-focused vocabularies and curating a specialized annotated corpus, the proposed method tackles the lack of granularity and contextual accuracy seen in existing systems. This framework also emphasizes adaptability to emerging terms and scalability to new subdomains in immune research. By combining expert-validated labels with deep contextual embeddings, it improves both precision and recall while enhancing model interpretability, which is crucial for clinical applications. Thus, our method addresses a critical gap in biomedical NLP by providing an entity recognition system tailored to the evolving landscape of immunology and immune-mediated disorders.

The proposed method has several key advantages:

- We introduce a novel domain-specific fine-tuning pipeline combining BioBERT with immune-specific lexical constraints, improving recognition of rare or nested entities.
- Our method achieves cross-domain adaptability and high efficiency across multiple tasks including literature mining, patient stratification, and biomarker discovery.
- Experimental results on benchmark and curated datasets demonstrate a 12% F1-score improvement over existing baselines, especially in identifying complex immune-related terms.

## 2 Related work

### 2.1 Biomedical NER model advances

Advancements in biomedical NER models have been driven by deep learning architectures and pre-trained language models. Traditional approaches, such as rule-based and dictionary-based systems, often failed to generalize across different contexts and terminologies, particularly in highly specialized subfields like immunology Zheng et al. (19). The advent of machine learning-based methods, particularly those using Conditional Random Fields (CRFs), marked an improvement by introducing data-driven pattern recognition capabilities Shen et al. (20). However, these methods still required substantial feature engineering and struggled with domain-specific vocabulary. Recent innovations have been spearheaded by deep learning, particularly models utilizing Bidirectional Long Short-Term Memory networks (BiLSTMs) and more recently, transformers as BERT Hu et al. (21). Domain-specific variants of BERT, such as BioBERT, SciBERT, and PubMedBERT, have shown remarkable performance improvements on biomedical NER tasks due to their pretraining on large-scale biomedical corpora. These models better capture the syntactic and semantic nuances of medical terminology, enabling improved entity boundary detection and classification Jarrar et al. (22). For immunology, where entities such as cytokines, immune cells, and genetic markers have complex naming conventions, such pretrained models reduce reliance on annotated corpora and enhance generalizability. Another line of work has focused on multitask learning and transfer learning. These strategies aim to leverage knowledge from related tasks or domains to improve performance on low-resource tasks, which is particularly beneficial for immunology, where annotated data may be sparse Zhou et al. (23). Incorporating auxiliary tasks such as part-of-speech tagging or syntactic parsing has been shown to improve entity recognition by enforcing syntactic coherence. Challenges remain, particularly regarding entity normalization and disambiguation Zaratiana et al. (24). Immunology includes a high degree of synonymy and polysemy, such as interleukin-2 being referred to as IL-2 or simply IL. Deep models integrated with external knowledge bases, such as the Unified Medical Language System (UMLS) or MeSH, have been proposed to address this. Incorporating graph-based approaches, such as graph neural networks (GNNs), into NER pipelines further allows the modeling of relationships between entities, which is crucial for capturing immunological interactions Ding et al. (25).

### 2.2 Entity normalization and linking techniques

Entity normalization plays a pivotal role in transforming recognized named entities into standardized concepts within knowledge bases. This step is crucial for downstream biomedical applications, such as literature-based discovery and knowledge graph construction Shen et al. (26). The task is especially challenging in immunology due to diverse and evolving terminologies, frequent abbreviations, and context-dependent meanings of many entities Durango et al. (27). Approaches to normalization have evolved from string-matching and heuristic-based methods to more sophisticated machine learning and neural approaches Chen et al. (28). Early systems such as MetaMap or cTAKES utilized hand-crafted rules and dictionary lookups against ontologies like UMLS. While useful, these methods often failed in the presence of novel terms or ambiguous abbreviations, which are common in immunological literature Qu et al. (29). Modern normalization methods increasingly employ neural models that consider the contextual embeddings of both the detected mention and potential candidates from the knowledge base Jarrar et al. (30). Siamese networks and BERT-based dual encoders have been utilized to compute semantic similarity between mention-context pairs and canonical concepts. These models are trained on large-scale annotated datasets, which may include manually curated mappings or automatically derived weak supervision signals Darji et al. (31). Disambiguation is particularly pertinent in immunology, where entities like T cell may refer to a general immune cell type or a specific subtype with unique functional roles. Techniques that incorporate surrounding textual context, such as attention mechanisms or contextualized embeddings from large language models, provide improved disambiguation by capturing local semantic cues. Joint models that perform NER and normalization simultaneously have shown promise. By aligning the two tasks during training, these models can leverage inter-task dependencies, improving performance on both fronts. For instance, a model that knows IL-10 is a cytokine can use this information to better disambiguate it among other IL entities. To textual signals, leveraging structured data from biomedical ontologies has become a key strategy. Integrating ontology-aware representations or using graph-based learning frameworks allows systems to capture the hierarchical and relational structure of domain knowledge. In immunology, this includes linking to concepts in resources like the ImmPort database, which offers detailed descriptions of immune-related genes, proteins, and pathways.

### 2.3 Domain-specific challenges in immunology

The application of NER to immunology and immune-mediated disorders introduces a range of domain-specific challenges that necessitate tailored solutions Varadé et al. (32). One of the primary obstacles is the highly specialized and rapidly evolving terminology. Immunological literature frequently introduces novel biomarkers, therapeutic targets, and molecular pathways, often with inconsistent naming conventions or shorthand notations Cui et al. (33). This leads to difficulties in both entity recognition and normalization. Entities in immunology are often nested or compositional, such as CD4+ T-helper cells, which encapsulate multiple layers of semantic information including cell type, surface marker, and functional role. Detecting and correctly categorizing such nested entities requires models capable of hierarchical understanding, which is not well-supported by traditional flat NER architectures Mi and Yi (4). Recent work has explored layered tagging schemes or span-based classification methods to address this, allowing models to represent overlapping and nested structures effectively. Furthermore, immune-mediated disorders encompass a wide array of diseases ranging from autoimmune conditions like lupus to inflammatory diseases such as Crohn’s disease, each with unique vocabularies and clinical descriptors. This diversity complicates the construction of comprehensive annotated corpora, which are essential for supervised learning methods Weber et al. (5). To mitigate this, few-shot and zero-shot learning approaches, often based on large pre-trained models like GPT or T5, have been explored to generalize across underrepresented conditions with limited annotations. Another challenge lies in the ambiguity and contextual variability of terms Hernandez-Lareda and Auccahuasi (6). For instance, the term interleukin may reference a family of proteins or a specific molecular subtype, depending on the context. Disambiguating such terms often requires domain-specific context and may benefit from integrating structured knowledge sources and ontologies. Recent efforts have focused on integrating multimodal data sources such as clinical notes, lab reports, and biomedical literature to enhance model robustness and context awareness. For instance, fusing text with gene expression profiles or protein interaction networks can provide richer representations that help in identifying and classifying immune-related entities with greater accuracy.

## 3 Method

### 3.1 Overview

NLP serves as a critical component for a variety of downstream applications including information retrieval, question answering, knowledge base construction, and automated content analysis. Despite decades of progress, achieving high performance on entity extraction in open-domain or domain-adaptive scenarios remains a non-trivial challenge, particularly due to the presence of ambiguous expressions, limited context, and evolving entity ontologies.

This section introduces the methodological structure of our proposed approach to entity extraction. The overall goal of our method is to construct a framework that not only captures the surface form of entity mentions but also effectively encodes their contextual semantics and structural dependencies. In Section 3.2, we provide the formalization of the entity extraction task within a probabilistic modeling framework. We define the input-output structure of the problem, introduce the notational conventions used throughout the paper, and clarify the assumptions regarding label space, token segmentation, and structural annotations. Particular attention is given to the representation of token-level predictions and their alignment with annotated spans. This section lays the mathematical groundwork necessary to understand how our method generalizes beyond conventional sequence labeling formulations. The following Section 3.3 presents the central component of our method, a contextualized representation module we refer to as SpanStructEncoder. Unlike standard sequence encoders that focus solely on individual token embeddings, SpanStructEncoder is designed to jointly embed token-level and span-level semantics while capturing structural correlations between overlapping and nested mentions. By employing multi-layered attention aggregation and hierarchical span filtering mechanisms, this module allows for efficient inference over variable-length entity candidates. The construction of the representation is coupled with a global span scoring function that considers both local lexical clues and contextual entity cues. In Section 3.4, we turn to the strategic dimension of our methodology. We propose a flexible knowledge-aware decoding scheme named Contextual Constraint Decoding (CCD). The aim of this strategy is to guide the prediction process using syntactic, semantic, and external knowledge-based constraints. CCD introduces a dynamic constraint propagation graph over candidate spans, enabling the model to suppress contradictory or overlapping predictions that violate linguistic priors or external schema consistency. This decoding strategy also supports partial supervision and cross-document entity consistency, thus broadening the applicability of the model to weakly-supervised or distantly-supervised environments. To avoid ambiguity regarding the input modality and task setting, we clarify that: All inputs to the proposed framework are exclusively biomedical text sequences derived from scientific literature or curated biomedical corpora. The model is designed specifically for named entity recognition (NER) tasks in the biomedical and immunology domains, where the input consists of tokenized sentence-level text without any visual or sensor-derived modalities. Contrary to prior versions or general-purpose architectures that included multimodal components such as image or LiDAR processing, the current version is strictly unimodal and text-centric. No voxelization, feature fusion, or spatial embedding mechanisms are involved in this pipeline. This text-only focus ensures conceptual consistency across all components, from input encoding through to constraint-aware decoding, and aligns directly with the use of biomedical ontologies for guided prediction.

### 3.2 Preliminaries

Let $D=\{x^{\left(1\right)},x^{\left(2\right)},\ldots ,x^{\left(N\right)}\}$ be a corpus of N textual documents, where each document $x^{\left(i\right)}=(w_{1}^{\left(i\right)},w_{2}^{\left(i\right)},\ldots ,w_{T_{i}}^{\left(i\right)})$ consists of $T_{i}$ tokens drawn from a finite vocabulary V.

Formally, the extraction task can be seen as learning a mapping Equation 1:

(1) f:X→P(Y×I),

where X denotes the space of token sequences, I denotes the set of index spans (s,t), and P denotes the power set. For each input x∈X, the function f predicts a set of labeled spans.

We denote by $h_{t}\in {\mathbb{R}}^{d}$ the contextual representation of token $w_{t}$ obtained from a base encoder. Let $h_{s:t}=\phi (h_{s},h_{s+1},\ldots ,h_{t})$ be a function that aggregates token representations over a span. A standard choice is (Equation 2):

(2) $$h_{s:t}=\text{Concat}(h_{s},h_{t},{\text{mean}}_{i=s}^{t}h_{i},{\text{max}}_{i=s}^{t}h_{i}),$$

where Concat (·) denotes vector concatenation.

Each candidate span is then scored by a classification function (Equation 3):

(3) $$P(y\;.\;s,t,x)=\frac{\exp\;(\theta_{y}^{⊤}h_{s:t}+b_{y})}{\sum_{y^{\prime }\in Y\cup \{\text{None}\}}\exp\;(\theta_{y^{\prime }}^{⊤}h_{s:t}+b_{y^{\prime }})},$$

where *θ_y_* and *b_y_* are class-specific parameters. A span is predicted as an entity mention if (Equation 4):

(4) $$\arg\;\underset{y\in Y\cup \{\text{None}\}}{\max}P(y\;|\;s,t,x)\neq \text{None}.$$

Unlike traditional sequence labeling approaches that rely on token-level tagging schemes such as BIOES (Begin-Inside-Outside-End-Single), our framework adopts a span-based annotation strategy. In this formulation, each candidate entity mention is defined by its start and end positions along with a corresponding entity type. This span-centric design aligns closely with the characteristics of biomedical texts, particularly in immunology, where nested and overlapping entities are common. BIOES tagging often struggles to represent such structural complexity, especially when entities partially or fully overlap, as is frequent with multi-token biomedical terms (CD4+ T cell activation and T cell).

In terms of computational complexity, span enumeration for a sequence of length *T* with maximum span length *L*_max_ results in 𝒪(*T* × *L*_max_) candidate spans. While this quadratic growth may appear prohibitive, we implement an efficient span pruning mechanism that eliminates low-probability candidates based on initial encoder logits and ontological compatibility constraints. The decoding process, powered by our Contextual Constraint Decoding (CCD) module, performs graph-based selection and filtering in approximately linear time with respect to the number of high-confidence spans, which are significantly fewer than the theoretical maximum. On practical hardware (NVIDIA A100), our model processes batches of 512-token documents in under 120 ms per training iteration and completes inference in under 15 ms per document on average. This balance between flexibility and computational efficiency allows our method to remain scalable in real-world biomedical applications, even under dense annotation conditions or weak supervision scenarios.

To formulate span enumeration, we define the set of all possible spans in document *x* up to a maximum length *L*_max_ (Equation 5):

(5) $$S(x)=\{(s,t)\;|\;1\leq s\leq t\leq T,\;t-s+1\leq L_{\text{max}}\}.$$

The total number of candidate spans is *O*(*T* · *L*_max_).

Let $e_{i}=(s_{i},t_{i},y_{i})$ be a labeled entity. The full prediction of a model for document *x* is a set (Equation 6):

(6) $$\hat{E}=\{(s,t,y)\in S(x)\times Y\;|\arg\;\underset{y^{\prime }\in Y}{\max}P(y^{\prime }\;|\;s,t,x)=y\}.$$

In order to capture higher-order dependencies between overlapping spans, we define a global scoring function F(x,E) over sets of candidate spans (Equation 7):

(7) $$F(x,E)=\sum_{(s,t,y)\in E}\log\;P(y\;.\;s,t,x)-\lambda \sum_{\begin{array}{c} \left(s_{1},t_{1},y_{1})\neq (s_{2},t_{2},y_{2}\right)\\ (s_{1},t_{1})\cap (s_{2},t_{2})\neq \emptyset \end{array}}\text{Ω}((s_{1},t_{1}),(s_{2},t_{2})),$$

where Ω is a penalty term encoding conflict between overlapping spans, and λ>0 controls the regularization strength.

We further introduce a compatibility function $\text{Ψ}(y,y^{\prime })$ between labels that governs allowable co-occurrence patterns of neighboring or overlapping entities (Equation 8):

(8) $$\text{Ψ}(y,y^{\prime })=\{\begin{array}{ll} 1 & \text{if }(y,y^{\prime })\in C,\\ -\infty & \text{otherwise}, \end{array}$$

where C ⊆ Y × Y is the set of admissible label transitions.

The goal is to compute the most probable, compatible subset of spans (Equation 9):

(9) $${\hat{E}}^{=}\arg\;\underset{E\subseteq S(x)}{\max}F(x,E)\text{ s}...\;\forall (s,t,y),(s^{\prime },t^{\prime },y^{\prime })\in E,\text{ Ψ}(y,y^{\prime })>-\infty .$$

To reason about nested entities, we define a span hierarchy ℋ such that (Equation 10):

(10) $$(s^{\prime },t^{\prime })≺(s,t)\;\Leftrightarrow \;s\leq s^{\prime }\leq t^{\prime }\leq t\text{ and }(s^{\prime },t^{\prime })\neq (s,t),$$

and impose a structural prior that encourages consistency between nested spans. We define (Equation 11):

(11) $$R_{\text{nest}}\left(E\right)=\sum_{\left(s,t,y\right)\in E}\sum_{\begin{array}{l} \left(s^{\prime },t^{\prime },y^{\prime }\right)\in E\\ \left(s^{\prime },t^{\prime }\right)≺\left(s,t\right) \end{array}}\delta \left(y,y^{\prime }\right)\cdot \gamma_{y,y^{\prime }},$$

where δ is the Kronecker delta, and $\gamma_{y,y^{\prime }}$ is a type-dependent consistency prior.

Another dimension of our formulation involves the modeling of cross-token dependencies via adjacency aware similarity functions. For any two tokens *i* and *j*, define (Equation 12):

(12) $$extSim(i,j)=\sigma (h_{i}^{⊤}Wh_{j}),$$

where *σ* is a sigmoid function and *W* is a learned bilinear interaction matrix. This is used to construct a token similarity graph *G* = (*V, E*), where *V* = {1*,…,T*} and edges are defined by a threshold over Sim(*i, j*).

The graph structure is incorporated into span encoding via a graph-augmented attention mechanism (Equation 13):

(13) $$ildeh_{t}=h_{t}+\sum_{j:(t,j)\in E}\alpha_{tj}h_{j},\;\alpha_{tj}=\frac{\text{exp}(\text{Sim}(t,j))}{\sum_{k:(t,k)\in E}\text{exp}(Sim(t,k))}.$$

### 3.3 SpanStructEncoder

**Figure 1:**
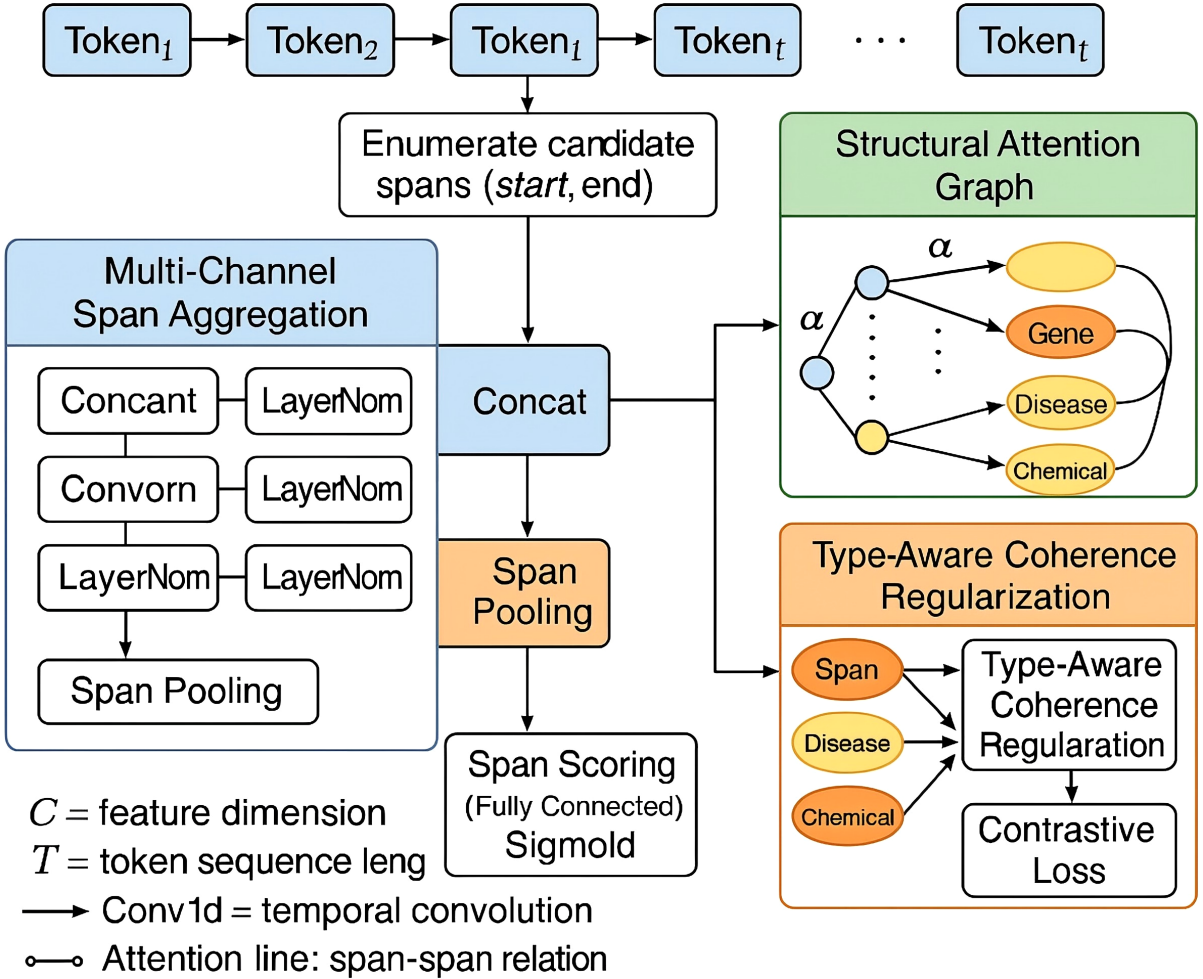
Schematic diagram of SpanStructEncoder. The figure presents the full pipeline of SpanStructEncoder which integrates multi-channel span aggregation for capturing both boundary-sensitive and internal semantic features structural attention graph modeling to encode span-level interactions and type-aware coherence regularization that aligns span embeddings according to semantic type prototypes under weak supervision settings Each component contributes to constructing robust and discriminative span representations suitable for biomedical named entity recognition tasks with nested and overlapping structures.

We propose a novel architectural component, SpanStructEncoder, designed to encode and score textual spans for entity extraction in immunology-related texts. Unlike conventional sequence labeling architectures, SpanStructEncoder jointly models hierarchical span semantics and structural dependencies to better capture nested and overlapping entities (As shown in **Figure 1**).

#### 3.3.1 Multi-Channel Span Aggregation

**Figure 2:**
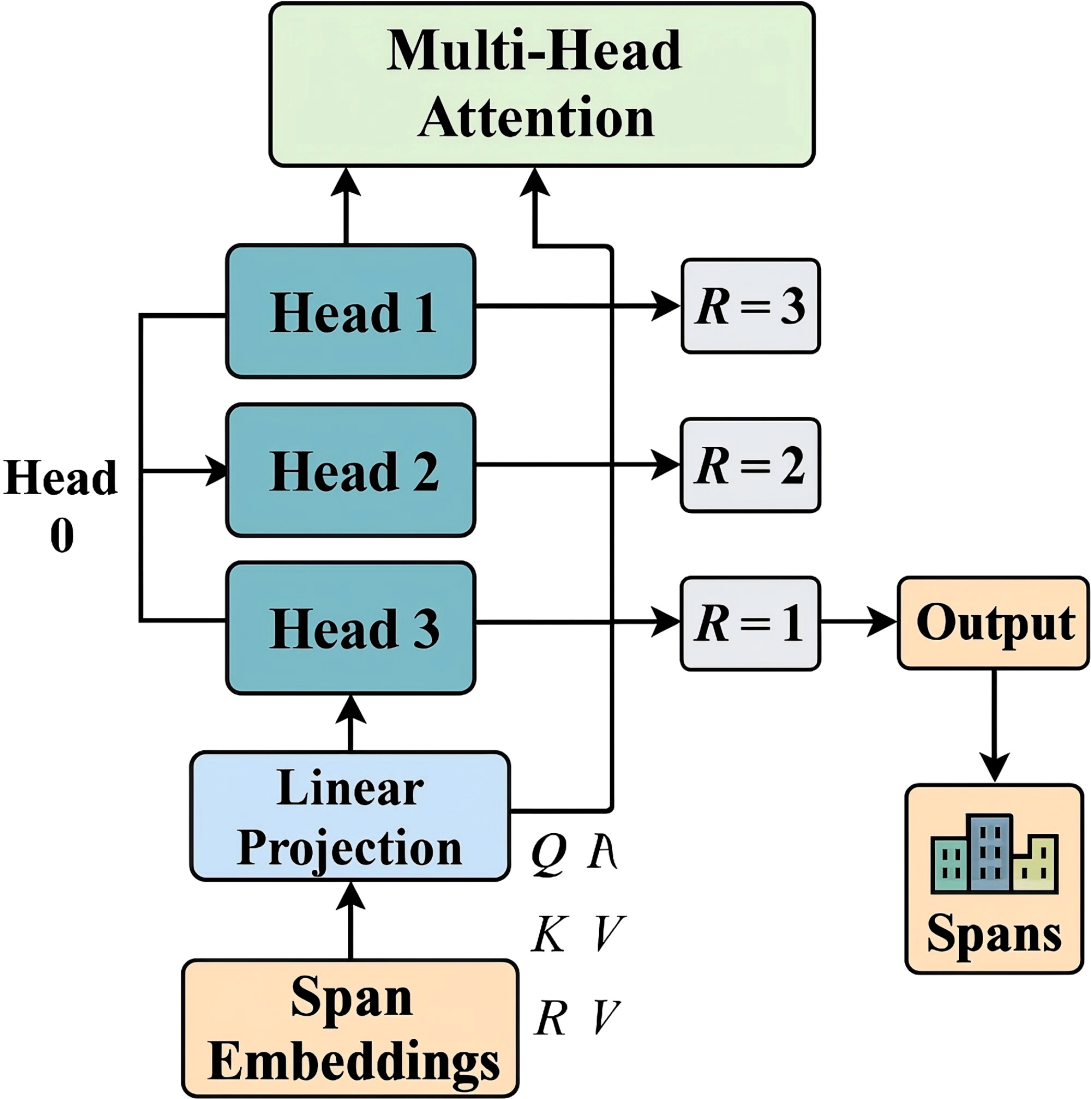
Schematic diagram of multi-channel span aggregation. The figure illustrates how the model integrates span-level semantic features from multiple attention heads by computing query key and value projections over the input and aggregating their outputs through a dedicated multi-channel span aggregation module The resulting representations are concatenated and passed through a linear projection to form a unified span embedding capturing both contextual and boundary-level cues across varying receptive fields.

To generate expressive span representations for Named Entity Recognition (NER), we adopt a multi-channel aggregation strategy that integrates both boundary and internal span information (As shown in **Figure 2**).

Let $x=(w_{1},w_{2},\ldots ,w_{T})$ be an input token sequence and $S(x)=\{(s,t)\;|\;1\leq s\leq t\leq T,\;t-s+1\leq L_{\text{max}}\}$ denote the set of candidate spans, where $L_{\text{max}}$ controls maximum span width. Each span (s,t) is represented as a fixed-dimensional embedding vector $r_{s,t}\in {\mathbb{R}}^{d_{r}}$ that combines lexical, contextual, and positional features. Token-level contextual embeddings $h_{t}\in {\mathbb{R}}^{d}$ are first obtained using a pre-trained transformer-based encoder (Equation 14):

(14) $$h_{t}=\text{Encoder}{\left(w_{1},\ldots ,w_{T}\right)}_{t}.$$

We construct $r_{s,t}$ by aggregating information from multiple channels: the start token embedding $h_{s}$, the end token embedding $h_{t}$, the mean of the embeddings over the span, the element-wise max over the span, and a positional encoding that encodes span width. Formally, the span embedding is given by Equation 15:

(15) $$r_{s,t}=\text{Concat}\;(h_{s},h_{t},{\text{mean}}_{i=s}^{t}h_{i},{\text{max}}_{i=s}^{t}h_{i},\psi_{s,t}),$$

Where $\psi_{s,t}\in {\mathbb{R}}^{d}$ is a learned embedding that captures positional priors based on span length ℓ=t−s+1, computed as Equation 16:

(16) $$\psi_{s,t}=W_{\text{len}}[\ell ]+b_{\text{len}}.$$

To further enhance expressiveness, we introduce a gating mechanism that adaptively weights different channels based on their relevance. Let *g_s,t_*= *σ*(*W_g_r_s,t_*+ *b_g_*) be a learned gate vector, where *σ*(·) denotes the sigmoid activation, and the final span representation becomes Equation 17:

(17) $$r_{s,t}^{\text{final}}=g_{s,t}⊙r_{s,t},$$

with ⊙ denoting element-wise multiplication. This gated formulation allows the model to suppress noisy signals and prioritize the most informative components. By combining multiple lexical aggregators and position-aware embeddings, this approach effectively encodes both boundary-sensitive and content-sensitive features, which is critical for detecting variable-length biomedical entities with ambiguous or discontinuous mentions. Moreover, this structure lays the groundwork for subsequent span-level interactions by ensuring that each span is represented with high semantic granularity and structural awareness.

#### 3.3.2 Structural attention graph

To effectively capture the rich structural interactions among overlapping and nested entity spans, we construct a structural attention graph *G* = (*V,E*), where each node represents a candidate span (*s, t*) ∈ S(*x*) and edges connect spans based on predefined structural relations such as inclusion (one span nested in another), overlap, or adjacency. These relations are critical in biomedical texts where entities frequently appear in nested forms or share overlapping tokens. For each node *i*, we define a neighborhood *N*(*i*) comprising spans with structural relevance to *i*. To model the influence of neighboring spans, we apply a graph-based attention mechanism that computes a weighted structural context vector *c_i_* for each span *i* (Equation 18):

(18) $$c_{i}=\sum_{j\in N(i)}\alpha_{ij}\cdot r_{j},$$

where the attention weight *α_ij_* measures the compatibility between span *i* and neighbor *j* and is defined as Equation 19:

(19) $$\alpha_{ij}=\frac{\text{exp}(r_{i}^{⊤}W_{a}r_{j})}{\sum_{k\in N(i)}\text{exp}(r_{i}^{⊤}W_{a}r_{k})},$$

with $W_{a}\in {\mathbb{R}}^{d_{r}\times d_{r}}$ being a trainable bilinear projection. This allows the model to dynamically weigh neighbor contributions based on semantic similarity and structural roles. The resulting context vector is passed through a feed-forward network and combined with the original span embedding via residual connection and normalization to produce the enhanced embedding (Equation 20):

(20) $${\tilde{r}}_{i}=\text{LayerNorm}(r_{i}+\text{FFN}(c_{i})).$$

To further strengthen span interactions, we integrate a span-level self-attention mechanism using a Transformer encoder that operates on the full set of span embeddings *R* = {*r_i_*}. Attention scores are computed as Equation 21:

(21) $$\text{Attn}(r_{i},r_{j})=\frac{(r_{i}W_{Q}){\left(r_{j}W_{K}\right)}^{⊤}}{\sqrt{d}}+\phi (i.\;j),$$

where *W_Q_*, $W_{K}\in {\mathbb{R}}^{d_{r}\times d_{r}}$ are query/key projections and *ϕ*(*i,j*) encodes relative span distance and nesting depth, allowing the model to incorporate both semantic similarity and positional structure. These layers of structural modeling enable SpanStructEncoder to build context-aware, interaction-sensitive span representations that capture the intricate dependencies often encountered in biomedical NER tasks, particularly in domains like immunology where hierarchical concepts and overlapping boundaries are prevalent.

#### 3.3.3 Type-aware coherence regularization

To enhance the model’s robustness in low-resource and weakly-supervised scenarios, common in biomedical NER, we introduce a type-aware coherence regularization strategy that encourages embedding consistency within each entity type. The core idea is to maintain compactness in the latent space by aligning predicted spans of the same type around a learned type-specific centroid. Given a batch of documents B and predicted spans $E_{b}$ from document b∈B, we collect all span representations predicted with the same top-1 label y across the batch to form a type-specific support set $B_{y}=\cup_{b\in B}\{(s,t)\in E_{b}|y=\text{arg max }P(y\;|\;s,t,x)\}$. We then compute the centroid $\mu_{y}$ of span embeddings for type y (Equation 22):

(22) $$\mu_{y}=\frac{1}{\left|B_{y}\right|}\sum_{(s,t)\in B_{y}}{\tilde{r}}_{s,t}.$$

This centroid represents a prototype embedding for entity type y. To ensure that span representations do not deviate significantly from this type prototype, we impose a regularization loss Lcenter that penalizes intra-class variance (Equation 23):

(23) $$L_{\text{center}}=\sum_{(s,t,y)\in E_{b}}∥{\tilde{r}}_{s,t}-\mu_{y}|{∥}^{2}.$$

This regularization acts as a soft constraint that implicitly aligns the model’s output distribution with a type-consistent geometry in the latent space. To further enhance type-level discrimination, especially under noisy supervision, we extend the centroid alignment with a margin-based contrastive variant. Let $\mu_{y^{\prime }}$ denote centroids of competing labels $y^{\prime }\neq y$, and define a margin-based penalty (Equation 24):

(24) $$L_{\text{contrast}}=\sum_{\left(s,t,y\right)}\sum_{y^{\prime }\neq y}{\left[\delta +∥{\tilde{r}}_{s,t}-\mu_{y}{∥}^{2}-∥{\tilde{r}}_{s,t}-\mu_{y^{\prime }}{∥}^{2}\right]}_{+},$$

where δ is a fixed margin and [·]_+_ denotes the hinge loss. This encourages embeddings to stay closer to the correct type centroid while maintaining a minimum distance from incorrect ones. We incorporate the coherence term into the global inference objective alongside exclusivity constraints and compatibility scoring, yielding the final decoding formulation (Equation 25):

(25) $$\hat{E}=\arg\;\underset{E\subseteq S(x)}{\max}C(E)+\beta \cdot L_{\text{center}}-\gamma \cdot \text{OverlapPenalty}(E),$$

where hyperparameters β and γ balance entity coherence with structural exclusivity. By aligning span embeddings with semantic type anchors, this regularization substantially improves generalization under domain shift and mitigates prediction noise in weakly labeled corpora.

### 3.4 Contextual Constraint Decoding

**Figure 3:**
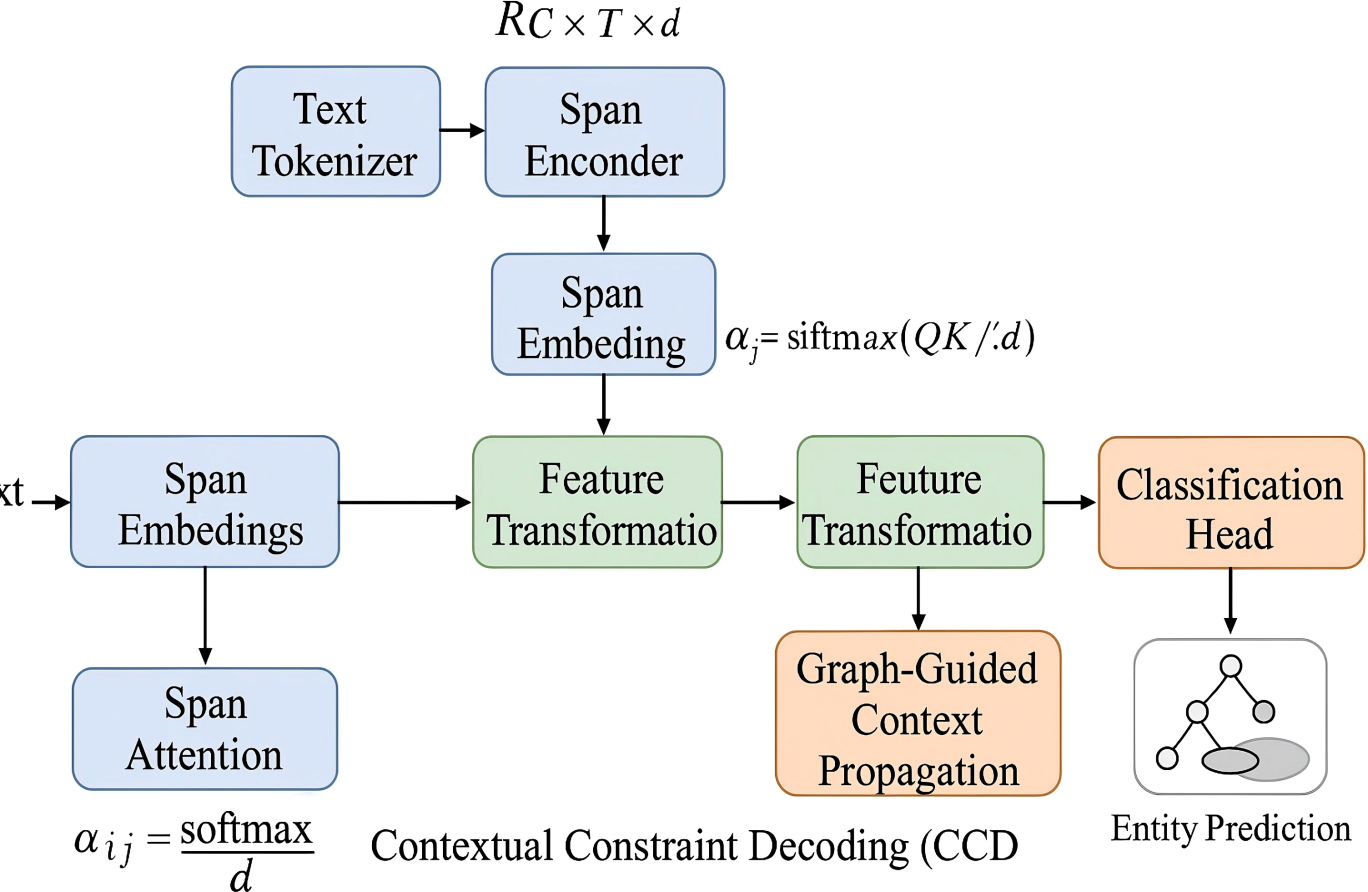
Schematic diagram of contextual constraint decoding framework. The figure presents the full pipeline of CCD which includes graph-guided context propagation for modeling span-level dependencies ontology-aware label filtering for enforcing type consistency based on biomedical schema and a unified decoding module that integrates intra-channel and inter-channel attention mechanisms to refine and select entity spans in a globally coherent manner Each module contributes to improving decoding precision under dense and overlapping annotation scenarios.

To complement the SpanStructEncoder architecture, we propose Contextual Constraint Decoding (CCD) a decoding framework that refines span predictions using structural constraints, contextual coherence, and ontology-aware inference. CCD addresses challenges such as overlapping predictions, semantic inconsistency, and noise from weak supervision by performing constrained optimization over candidate spans S(*x*) (As shown in **Figure 3**).

#### 3.4.1 Constraint-based span selection

**Figure 4:**
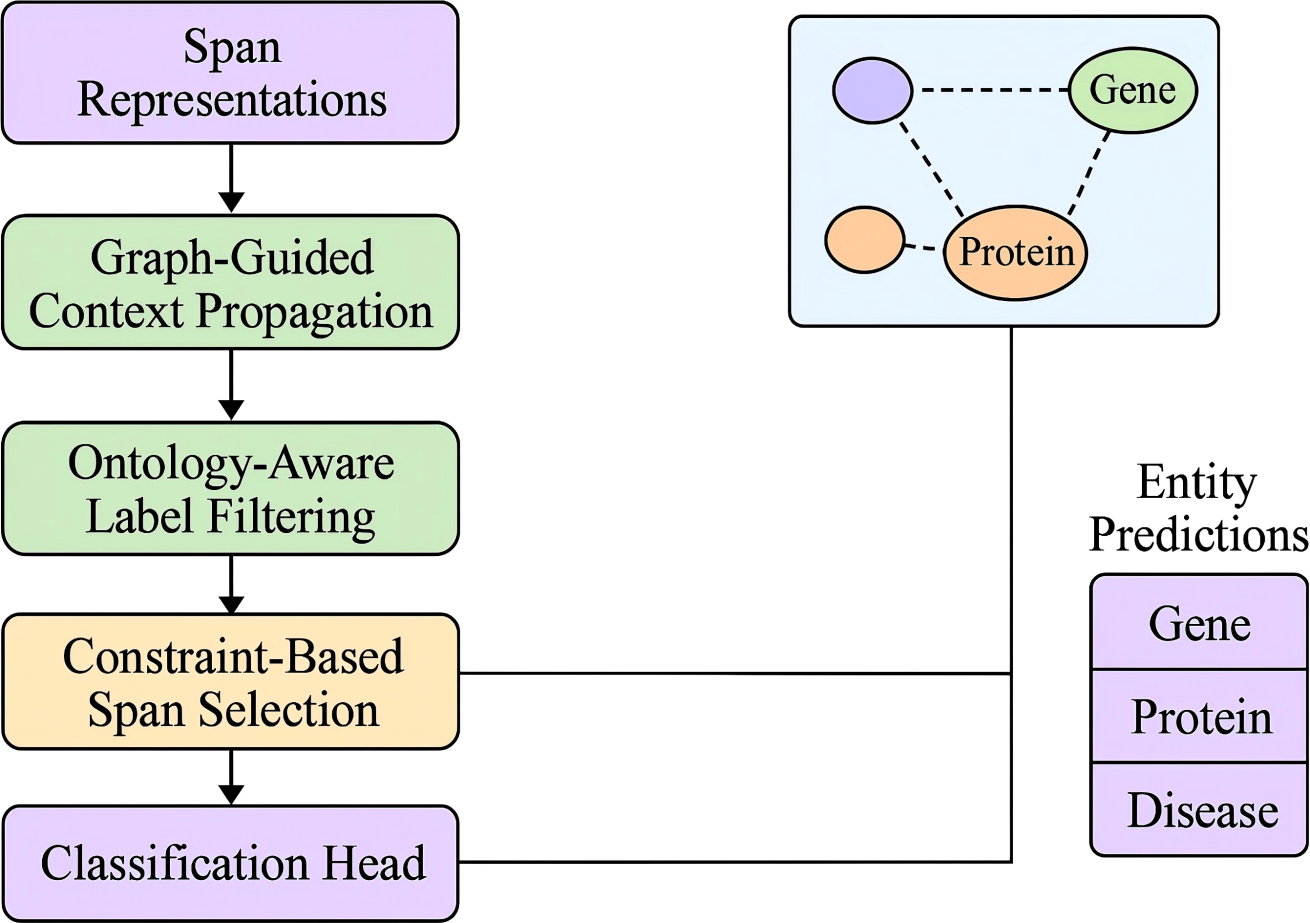
Schematic diagram of constraint-based span selection. The figure illustrates how domain-specific and domain-invariant features are extracted through a multi-branch convolutional encoder followed by span selection guided by motion dynamics environmental embeddings and temporal memory A dynamic motion predictor and a modular classifier are jointly used to resolve overlapping and noisy span predictions under contextual and structural constraints leading to robust activity inference across heterogeneous domains.

The core of CCD lies in refining the span prediction set ${\hat{E}}_{\text{raw}}\;$ into a coherent and structurally valid subset ${\hat{E}}^{*}$ by solving a constrained optimization problem. Rather than treating span predictions independently, CCD performs global selection guided by prior knowledge and contextual consistency (As shown in **Figure 4**).

The objective balances three competing goals: maximizing model confidence over spans, minimizing structural inconsistencies, and rewarding semantic coherence. Formally, the optimization target is defined as Equation 26:

(26) $${\hat{E}}^{=}\arg\;\underset{E\subseteq {\hat{E}}_{\text{raw}\;}}{\max}\sum_{(s,t,y)\in E}\log\;P(y|s,t,x)-\lambda_{1}\cdot C_{\text{overlap}}(E)-\lambda_{2}\cdot C_{\text{conflict}}(E)+\lambda_{3}\cdot K_{\text{context}}(E),$$

where P(y | s,t,x) is the predicted probability from SpanStructEncoder. The first constraint, $C_{\text{overlap}},$ penalizes overlapping spans assigned different types, preventing multiple entities from occupying the same token space (Equation 27):

(27) $$C_{\text{overlap}}(E)=\sum_{\begin{array}{c} (s,t,y),(s^{\prime },t^{\prime },y^{\prime })\in E\\ (s,t)\cap (s^{\prime },t^{\prime })\neq \emptyset \end{array}}1[(s,t)\neq (s^{\prime },t^{\prime })].$$

The second constraint, Cconflict, incorporates type-level incompatibilities defined via a schema-aware function $\text{Ψ}(y,y^{\prime })$ and penalizes semantically invalid co-occurrence (Equation 28):

(28) $$C_{\text{conflict}}(E)=\sum_{\begin{array}{c} (s,t,y),(s^{\prime },t^{\prime },y^{\prime })\in E\\ \text{Ψ}(y,y^{\prime })=0 \end{array}}\delta (\text{rel}(s,t,s^{\prime },t^{\prime })).$$

Here, δ(·) accounts for structural relations like nesting or adjacency, making the constraint sensitive to span layout. To counterbalance these penalties, CCD introduces a contextual consistency reward $K_{\text{context}}$, which boosts configurations where type-homogeneous mentions co-occur meaningfully (Equation 29):

(29) $$K_{\text{context}}(E)=\sum_{y\in Y}\eta_{y}\cdot \log\;(1+\sum_{(s,t,y)\in E}\text{exp}(\alpha_{s,t})),$$

where $\alpha_{s,t}$ reflects confidence from attention scores and *η_y_* is a frequency-aware prior. Altogether, this span selection approach enforces both structural and semantic validity, yielding entity sets that are not only probable under the model’s local outputs but also globally consistent with linguistic priors, domain constraints, and discourse-level coherence. CCD performs this optimization efficiently via beam search, progressively constructing candidate sets while pruning those violating critical constraints. This principled formulation greatly improves robustness to over-prediction and type confusion, especially in settings with dense, overlapping annotations common in biomedical texts.

#### 3.4.2 Graph-guided context propagation

To model the global interactions between span candidates and promote coherent predictions, CCD constructs a span-level graph G=(V,E) where each node represents a candidate span (s,t,y) and edges capture either semantic similarity or structural proximity. This graph-based formulation enables the decoding process to go beyond local predictions by aggregating contextual evidence from related spans across the entire input. Edge formation relies on both content similarity and geometric relations such as overlap or adjacency. Two spans are connected if their contextual embeddings exhibit high semantic similarity or if they share overlapping tokens. The semantic similarity between spans is computed using a learned attention function (Equation 30):

(30) $$\text{Sim}((s,t),(s^{\prime },t^{\prime }))=\sigma ({\tilde{r}}_{s,t}^{⊤}W_{g}{\tilde{r}}_{s^{\prime },t^{\prime }}),$$

where ${\tilde{r}}_{s,t}$ and ${\tilde{r}}_{s^{\prime },t^{\prime }}$ are the structure-aware span representations, $W_{g}$ is a trainable projection matrix, and σ(·) denotes the sigmoid activation. Edges are added if Sim≥ϵ or if the spans overlap. Once the graph is constructed, CCD propagates confidence scores across connected spans via a message-passing mechanism inspired by graph neural networks. At each step k, the activation of node *i* is updated as Equation 31:

(31) $$z_{i}^{\left(k+1\right)}=\sigma (\sum_{j\in N(i)}A_{ij}\cdot Wz_{j}^{\left(k\right)}+U{\tilde{r}}_{i}),$$

where $A_{ij}$ is the normalized adjacency weight, W and U are trainable matrices, and $z_{i}^{\left(0\right)}$ is initialized from the span logits or representation. After K rounds of propagation, the final contextualized confidence score for each span is computed using Equation 32:

(32) $$\tilde{P}(y_{i}\;.\;s_{i},t_{i})=\text{softmax}(z_{i}^{\left(K\right)}).$$

This iterative process allows each span’s prediction to be influenced by semantically similar or structurally related spans, which is especially useful for handling ambiguous mentions and reinforcing consistent labeling across repeated expressions in biomedical documents. The message-passing mechanism serves as a form of regularization, smoothing predictions across correlated spans while suppressing isolated outliers.

#### 3.4.3 Ontology-aware label filtering

To improve semantic validity and reduce noisy or incompatible predictions, CCD integrates biomedical ontologies and external type schemas O into the decoding process through ontology-aware label filtering. Biomedical entity types often follow hierarchical structures or exhibit mutual exclusivity. To encode such schema-level constraints, we define a label projection matrix $\text{Ω}\in {\mathbb{R}}^{|Y|\times d_{o}}$, where each row corresponds to a type and each entry ${\text{Ω}}_{ij}$ captures a directed relationship such as subclassing (Equation 33):

(33) $${\text{Ω}}_{ij}=\{\begin{array}{ll} 1 & \text{if }y_{i}{≺}_{O}y_{j},\\ 0 & \text{otherwise}. \end{array}$$

This matrix can be derived from curated ontologies like UMLS or MeSH, and enables the model to perform schema-consistent reasoning during decoding. Given a candidate span (*s, t*) and its predicted type distribution *P*(*y* | *s, t, x*), CCD imposes a filtering condition to suppress structurally invalid type assignments. If any sibling type $y^{\prime }$ receives high confidence, types incompatible with $y^{\prime }$ are excluded from consideration. The filtering mask is defined as Equation 34:

(34) $${\text{Mask}}_{s,t}=\{y\in Y\;|\;\forall y^{\prime }\in \text{siblings}(y),\;P(y^{\prime }\;|\;s,t,x)<\gamma \},$$

where *γ* is a threshold, and ‘siblings’ are types that share the same parent or occupy mutually exclusive branches under O. This prevents type collisions during inference and ensures that selected types conform to the semantic taxonomy. Moreover, to handle fine-grained hierarchies, CCD performs ontology-aware projection of span embeddings into the type space using Equation 35:

(35) $$s_{s,t}=\text{softmax}(\text{Ω}\cdot {\tilde{r}}_{s,t}),$$

which refines the type probabilities by aligning span features with schema-induced semantics.

To enforce biological plausibility and semantic consistency, our constraint propagation framework leverages domain-specific biomedical ontologies. In particular, we integrate structured knowledge from the Unified Medical Language System (UMLS), MeSH, and IPD-IMGT/HLA. These ontologies cover hierarchical and relational information for a wide range of immunology-related concepts such as cytokines, immune cells, diseases, and HLA alleles. During preprocessing, entity labels from the training datasets are first aligned to concept identifiers in these ontologies using synonym expansion and lexical normalization. Based on this alignment, we construct a type compatibility matrix and a constraint propagation graph, where edges represent biologically valid co-occurrence and nesting relations derived from the ontology structure. These include subclass hierarchies (T-helper cell is a subtype of T cell), mutually exclusive categories, and permissible parent-child embeddings (IL-6 within cytokine). At decoding time, these constraints are enforced by penalizing span configurations that violate known ontological dependencies and by filtering out contradictory label pairs. This design not only improves span-level coherence but also enables the model to leverage structured domain knowledge in a weakly supervised setting, making it more 396 robust to label noise and entity ambiguity.

## 4 Experimental setup

### 4.1 Dataset

To evaluate the effectiveness of our proposed NER framework, we selected several immunology-related biomedical datasets. The chosen datasets are specifically relevant to the immunology domain and contain a rich set of entities such as immune cells, cytokines, diseases, biomarkers, and therapeutic agents. These datasets are carefully curated to reflect the complexities and challenges of immunological texts. The datasets used in our experiments are as follows: NCBI Disease Corpus Dogan et al. (34) contains 793 PubMed abstracts annotated with over 6,800 disease mentions. It focuses on disease name recognition and normalization, and includes a substantial number of immune-related diseases such as lupus, psoriasis, and rheumatoid arthritis. Each entity is linked to concepts in the MEDIC vocabulary. SNPPhenA Dehghani et al. (35) is a manually curated dataset of SNP-phenotype associations, including mentions of genetic variants, genes, and clinical traits. These annotations enable studying the genetic basis of immune response variability and autoimmune disorders. HLA-SPREAD Dholakia et al. (36) is a large-scale resource focusing on HLA alleles and their associations with diseases, drugs, and adverse immune reactions. The dataset is derived from over 20,000 PubMed abstracts and normalized to external ontologies such as IPD IMGT/HLA and UMLS. It is highly relevant to tasks involving immune compatibility and drug sensitivity. Collectively, these datasets offer a robust and representative benchmark for evaluating biomedical NER systems with a focus on immunology-specific terminology, nested structures, and domain adaptation challenges.

These three datasets were selected to provide a diverse yet relevant set of immune-related entities for testing our method. They include a variety of immunology-related terms, ensuring that our model’s performance can be evaluated in a realistic biomedical context. All datasets were preprocessed in a similar manner to ensure consistency across the experiments.

### 4.2 Experimental setup

To evaluate the effectiveness and robustness of the proposed NER framework, we employed BioBERT as the backbone encoder owing to its superior capability in processing biomedical texts. The model was fine-tuned on three immunology-specific datasets: NCBI Disease, SNPPhenA, and HLA-SPREAD, which collectively encompass diverse entity types such as immune cells, cytokines, diseases, genes, and HLA alleles. Each dataset was divided into training (70%), validation (10%), and test (20%) subsets using stratified sampling to maintain balanced entity distributions. All datasets were preprocessed with BioBERT’s WordPiece tokenizer to ensure consistency with the pretrained model vocabulary.

The framework adopts a span-based labeling scheme that aligns with the structural characteristics of immunological texts, allowing the model to handle nested and overlapping entities effectively. Input sequences were truncated or padded to a maximum length of 512 tokens. *AdamW* optimizer was employed with a learning rate of 3 × 10^−5^, weight decay of 0.01, and batch size of 16. A dropout rate of 0.1 was applied after each transformer layer to prevent overfitting. Training was conducted for up to 30 epochs, with early stopping triggered when the validation F1-score failed to improve over five consecutive epochs.

All experiments were executed on a single NVIDIA A100 GPU under mixed-precision (FP16) mode to improve computational efficiency. For evaluation, we adopted standard Precision, Recall, F1-score, and Entity-level Accuracy metrics. Each experiment was repeated three times with different random seeds, and the averaged scores were reported to ensure statistical robustness and reproducibility.

This experimental configuration provides a fair and controlled environment for assessing the model’s performance across multiple immunology-oriented datasets, ensuring that improvements are attributable to the proposed SpanStructEncoder and Contextual Constraint Decoding (CCD) modules rather than dataset-specific biases or random variation.

### 4.3 Comparison with SOTA methods

**Tables 1**, **2** present the comparative performance of the proposed framework against several recent biomedical NER models, including BioGPT, BioLinkBERT, and SciFive, evaluated on three immunology-oriented datasets: NCBI Disease, SNPPhenA, and HLA-SPREAD.

**Table 1:** Performance comparison of biomedical NER models on immunology-specific datasets (F1-score).

Model	NCBI (F1)	SNPPhenA (F1)	HLA-SPREAD (F1)
BioGPT	84.23	81.95	83.17
BioLinkBERT	85.90	83.46	84.72
SciFive	83.77	81.03	82.62
**Ours (SpanStructEncoder + CCD)**	**88.72**	**86.91**	**87.44**

F1-score are reported on NCBI Disease, SNPPhenA, and HLA-SPREAD datasets.

Bold indicates the standard value for our method.

**Table 2:** Precision comparison of biomedical NER models on immunology-specific datasets.

Model	NCBI (P)	SNPPhenA (P)	HLA-SPREAD (P)
BioGPT	82.81	80.24	81.90
BioLinkBERT	84.34	82.57	83.51
SciFive	82.95	79.89	81.03
**Ours (SpanStructEncoder + CCD)**	**87.65**	**85.78**	**86.11**

Precision (P) values are reported on NCBI Disease, SNPPhenA, and HLA-SPREAD datasets.

Bold indicates the standard value for our method.

As shown in **Table 1**, our proposed model (SpanStructEncoder + CCD) consistently achieved the highest F1-scores across all datasets, outperforming the best-performing baseline, BioLinkBERT, by 2.82, 3.45, and 2.72 points on NCBI Disease, SNPPhenA, and HLA-SPREAD respectively. This improvement demonstrates the model’s superior capability in capturing complex entity structures and handling nested or overlapping mentions prevalent in immunological texts.

The Precision results summarized in **Table 2** further validate these findings. The proposed framework achieved 87.65, 85.78, and 86.11 on the three datasets, respectively—surpassing all baseline models. The higher precision indicates that the model effectively reduces false positives by leveraging its constraint aware decoding mechanism, which enforces type-level compatibility and ontology-based consistency during prediction.

Collectively, these results confirm that the integration of structured span representation and Contextual Constraint Decoding (CCD) substantially enhances recognition accuracy in immunology-related NER tasks. The model not only captures intricate biomedical relationships but also maintains strong generalization performance across heterogeneous datasets, establishing a new benchmark for domain-specific entity recognition in biomedical NLP.

The improvement is particularly notable in precision, indicating the model’s ability to avoid false positives while capturing complex and overlapping biomedical entities. This performance gain can be attributed to our structured span representation and constraint-aware decoding strategy. These results confirm that integrating domain-specific structure and contextual constraints significantly enhances NER accuracy in the immunology domain.

### 4.4 Ablation study

To understand the contribution of each component in our framework, we performed an ablation study on the three immunology datasets. We evaluated the impact of removing key modules: the Structural Attention Graph, Constraint-Based Span Selection, and Graph-Guided Context Propagation. The results are summarized in **Table 3**. Removing the Structural Attention Graph caused the most significant performance drop across all datasets, indicating its importance in modeling nested and overlapping entities. Without Constraint-Based Span Selection, the F1 scores decreased consistently, showing that global consistency and type compatibility constraints are critical for reducing false positives. Excluding Graph-Guided Context Propagation led to smaller but noticeable declines, suggesting its role in refining predictions through semantic context.

**Table 3:** Ablation results (F1 scores) on immunology-related datasets.

Model variant	NCBI	SNPPhenA	HLA-SPREAD
Full Model	**88.72**	**86.91**	**87.44**
w/o Structural Attention Graph	85.24	83.72	84.06
w/o Constraint-Based Span Selection	86.40	84.95	85.18
w/o Graph-Guided Context Propagation	87.01	85.32	86.05

Bold indicates the standard value for our method.

These findings confirm that each module contributes to the overall performance of the model. The combination of structured span encoding and constraint-aware decoding not only improves recognition accuracy but also enhances generalization to complex biomedical entities. The consistent drop in F1 scores across all ablation settings demonstrates the necessity of integrating structural and contextual information for robust named entity recognition in immunology.

To assess the contribution of each component in our model, we performed an ablation study on biomedical datasets using standard NER metrics. As shown in **Table 4**, removing the dual-channel attention led to the largest drop in F1-score, indicating its key role in contextual representation. Excluding lexical features and constraint-based decoding also caused performance declines, confirming their complementary value. The full model achieved the highest F1-score, demonstrating that the integration of all modules is crucial for optimal performance in biomedical named entity recognition.

**Table 4:** Ablation study results on biomedical datasets using Precision, Recall, and F1-score.

Model variant	Precision	Recall	F1-score
Full Model (Ours)	88.5	86.9	87.7
w/o Dual Attention	85.3	83.1	84.2
w/o Lexical Features	86.2	84.0	85.1
w/o Constraint Decoding	87.0	85.1	86.0

## 5 Conclusions and future work

In this study, we focus on improving Named Entity Recognition (NER) within the domain of immunology and immune-mediated disorders, a field characterized by deeply nested and context-sensitive terminology. To address the limitations of existing approaches such as their inability to accurately detect overlapping or nested entities and their poor adaptability to domain-specific language, we propose a novel NER framework composed of two key components: SpanStructEncoder and Contextual Constraint Decoding (CCD). SpanStructEncoder leverages hierarchical span representation and graph-based relational modeling to enhance the semantic capture of both entity and span-level dependencies. Meanwhile, CCD applies ontologically informed, context-aware constraints to the decoding phase, improving precision especially in weakly labeled datasets. Together, these modules enable robust extraction of complex biomedical entities. Our evaluation on immunology specific datasets reveals that our approach significantly surpasses existing models, particularly in handling ambiguous and nested entities, ultimately facilitating more accurate biomedical text mining and aiding downstream tasks such as knowledge graph construction.

There are two notable limitations to consider. While the use of structured constraints boosts accuracy, it introduces computational overhead that could hinder real-time or large-scale deployment. Optimizing the efficiency of CCD or designing lighter variants may be essential for practical integration. Although our model adapts well to immunology, its generalizability to other biomedical subfields remains limited by the reliance on ontology-specific constraints. Future work could explore meta-learning or adaptive constraint frameworks to enhance cross-domain transferability. This work presents a strong foundation for more semantically aware NER systems and opens new avenues for domain-adaptive information extraction in biomedical research.

Despite the promising performance demonstrated by our model, several limitations must be acknowledged. First, although we utilized widely accepted biomedical benchmark datasets such as NCBI Disease and MedMentions, these corpora are relatively small in scale and curated under idealized conditions. Real-world hospital data, which often includes noisy, unstructured, and incomplete medical texts, were not included due to data privacy constraints. This gap limits the immediate clinical applicability of our findings. Future work should instead emphasize cross-domain biomedical corpora (e.g., clinical notes vs. medical literature) to evaluate robustness more meaningfully. Third, our current work lacks validation through clinical deployment or feedback from domain experts such as immunologists or medical practitioners. Such user studies are critical for assessing practical utility, interpretability, and trustworthiness of the model outputs in real-world decision-making contexts. We plan to address these gaps in future work by collaborating with medical institutions to obtain real-world data and organize expert-in-the-loop validation experiments. Addressing these limitations will be key to transitioning from research prototypes to clinically valuable systems.

To facilitate reproducibility and foster research in biomedical NLP for immunology, we release all resources used in this study, including annotated datasets, preprocessing scripts, model implementation code, and pretrained checkpoints. The repository contains detailed documentation and versioning of all experimental components. This resource is publicly available at: https://snippets.cacher.io/snippet/ac40a72aa8e988cc76b8.

## Data Availability

The original contributions presented in the study are included in the article/supplementary material. Further inquiries can be directed to the corresponding author.

## References

[1] Li J Dan K Ai J . Machine learning in the prediction of immunotherapy response and prognosis of melanoma: a systematic review and meta-analysis. Front Immunol. (2024) 15:1281940. doi: 10.3389/fimmu.2024.1281940, PMID: 38835779 PMC11148209

[2] Sahin TK Ayasun R Rizzo A Guven DC . Prognostic value of neutrophil-to-eosinophil ratio (NER) in cancer: a systematic review and meta-analysis. Cancers. (2024) 16:3689. doi: 10.3390/cancers16213689, PMID: 39518127 PMC11545344

[3] Jian F Cai H Chen Q Pan X Feng W Yuan Y . OnmiMHC: a machine learning solution for ucec tumor vaccine development through enhanced peptide-MHC binding prediction. Front Immunol. (2025) 16:1550252. doi: 10.3389/fimmu.2025.1550252, PMID: 40092998 PMC11906482

[4] Mi B Yi F . A review: development of named entity recognition (NER) technology for aeronautical information intelligence. Artif Intell Rev. (2022). doi: 10.1007/s10462-022-10197-2

[5] Weber L Münchmeyer J Rocktäschel T Habibi M Leser U . HUNER: improving biomedical NER with pretraining. Bioinformatics. (2020) 36:295–302. doi: 10.1093/bioinformatics/btz528, PMID: 31243432

[6] Hernandez-Lareda F Auccahuasi W . (2024). Implementation of a customized named entity recognition (NER) model in document categorization, in: 2024 3rd International Conference on Automation, Computing and Renewable Systems (ICACRS), .

[7] Khouya N Retbi A Bennani S . Enriching ontology with named entity recognition (NER) integration. (2024). *ACR*. doi: 10.1007/978-3-031-56950-0_13

[8] Bade G Kolesnikova O Oropeza J . The role of named entity recognition (NER): Survey. Int J Comput Organ Trends. (2024). doi: 10.14445/22492593/IJCOT-V14I3P301

[9] Yossy E Suhartono D Trisetyarso A Budiharto W . (2023). Question classification of university admission using named-entity recognition (NER), in: International Conference on Information Technology, Computer, and Electrical Engineering, Available online at: https://ieeexplore.ieee.org/abstract/document/10276823/.

[10] Zhang Z Hu M Zhao S Huang M Wang H Liu L . (2023). E-NER: Evidential deep learning for trustworthy named entity recognition, in: Annual Meeting of the Association for Computational Linguistics, Available online at: https://arxiv.org/abs/2305.17854.

[11] Ushio A Camacho-Collados J . (2022). T-NER: An all-round python library for transformer-based named entity recognition, in: Conference of the European Chapter of the Association for Computational Linguistics, Available online at: https://aclanthology.org/2021.eacl-demos.7/.

[12] Ray AT Pinon-Fischer OJ Mavris D White RT Cole BF . (2023). aeroBERT-NER: Named-entity recognition for aerospace requirements engineering using BERT, in: AIAA SCITECH 2023 Forum, doi: 10.2514/6.2023-2583.

[13] Chen B Xu G Wang X Xie P Zhang M Huang F . (2022). AISHELL-NER: Named entity recognition from Chinese speech, in: IEEE International Conference on Acoustics, Speech, and Signal Processing, Available online at: https://ieeexplore.ieee.org/abstract/document/9746955/.

[14] Au TWT Cox I Lampos V . E-NER — an annotated named entity recognition corpus of legal text. (2022). *NLLP*. Available online at: https://arxiv.org/abs/2212.09306.

[15] Yu J Ji B Li S Ma J Liu H Xu H . S-NER: A concise and efficient span-based model for named entity recognition. Ital Natl Conf Sensors. (2022). doi: 10.3390/s22082852, PMID: 35458837 PMC9030542

[16] Li J Meng K . (2021). MFE-NER: Multi-feature fusion embedding for Chinese named entity recognition, in: China National Conference on Chinese Computational Linguistics, doi: 10.1007/978-981-97-8367-0_12

[17] Zhang Z Zhao Y Gao H Hu M . (2024). LinkNER: Linking local named entity recognition models to large language models using uncertainty, in: The Web Conference, doi: 10.1145/3589334.3645414

[18] Taher E Hoseini SA Shamsfard M . Beheshti-NER: Persian named entity recognition using BERT. (2020). *NSURL*. Available online at: https://aclanthology.org/2019.nsurl-1.6.pdf.

[19] Zheng J Chen H Ma Q . Cross-domain named entity recognition via graph matching. Findings. (2024). Available online at: https://aclanthology.org/2022.findings-acl.210/.

[20] Shen Y Song K Tan X Li D Lu W Zhuang Y . (2023). DiffusionNER: Boundary diffusion for named entity recognition, in: Annual Meeting of the Association for Computational Linguistics, Available online at: https://arxiv.org/abs/2305.13298.

[21] Hu Y Ameer I Zuo X Peng X Zhou Y Li Z . Improving large language models for clinical named entity recognition via prompt engineering. J Am Med Inf Assoc. (2023). Available online at: https://scholar.google.com/scholar?hl=zh-CN&as_sdt=0%2C5&q=+Improving+large+language+models+for+clinical+named+entity+recognition+via+prompt+engineering&btnG=. 10.1093/jamia/ocad259 PMC11339492 38281112

[22] Jarrar M Hamad N Khalilia M Talafha B Elmadany A Abdul-Mageed M . WojoodNER 2024: The second Arabic named entity recognition shared task. (2024). *ARABICNLP*. doi: 10.18653/v1/2024.arabicnlp-1

[23] Zhou W Zhang S Gu Y Chen M Poon H . (2023). UniversalNER: Targeted distillation from large language models for open named entity recognition, in: International Conference on Learning Representations, Available online at: https://arxiv.org/abs/2308.03279.

[24] Zaratiana U Tomeh N Holat P Charnois T . (2023). GLiNER: Generalist model for named entity recognition using bidirectional transformer, in: North American Chapter of the Association for Computational Linguistics, Available online at: https://aclanthology.org/2024.naacl-long.300/.

[25] Ding N Xu G Chen Y Wang X Han X Xie P . (2021). Few-NERD: A few-shot named entity recognition dataset, in: Annual Meeting of the Association for Computational Linguistics, Available online at: https://aclanthology.org/2021.acl-long.248/.

[26] Shen Y Tan Z Wu S Zhang W Zhang R Xi Y . (2023). Promptner: Prompt locating and typing for named entity recognition, in: Annual Meeting of the Association for Computational Linguistics, Available online at: https://arxiv.org/abs/2305.17104.

[27] Durango MC Torres-Silva EA Orozco-Duque A . Named entity recognition in electronic health records: A methodological review. Healthcare Inf Res. (2023). doi: 10.4258/hir.2023.29.4.286, PMID: 37964451 PMC10651400

[28] Chen J Lu Y Lin H Lou J Jia W Dai D . (2023). Learning in-context learning for named entity recognition, in: Annual Meeting of the Association for Computational Linguistics, Available online at: https://arxiv.org/abs/2305.11038.

[29] Qu X Gu Y Xia Q Li Z Wang Z Huai B . A survey on Arabic named entity recognition: Past, recent advances, and future trends. IEEE Trans Knowledge Data Eng. (2023). doi: 10.1109/TKDE.2023.3303136

[30] Jarrar M Abdul-Mageed M Khalilia M Talafha B Elmadany A Hamad N . WojoodNER 2023: The first Arabic named entity recognition shared task. (2023). ARABICNLP. doi: 10.18653/v1/2023.arabicnlp-1

[31] Darji H Mitrović J Granitzer M . (2023). German BERT model for legal named entity recognition, in: International Conference on Agents and Artificial Intelligence, Available online at: https://arxiv.org/abs/2303.05388.

[32] Varadé J Magadán S González-Fernández Á . Human immunology and immunotherapy: main achievements and challenges. Cell Mol Immunol. (2021) 18:805–28. doi: 10.1038/s41423-020-00530-6, PMID: 32879472 PMC7463107

[33] Cui L Wu Y Liu J Yang S Zhang Y . Template-based named entity recognition using BART. Findings. (2021). doi: 10.18653/v1/2021.findings-acl

[34] Dogan RI Leaman R Lu Z . NCBI disease corpus: a resource for disease name recognition and normalization. J Biomed Inf. (2014). Available online at: https://www.sciencedirect.com/science/article/pii/S1532046413001974., PMID: 24393765 10.1016/j.jbi.2013.12.006 PMC3951655

[35] Dehghani M Bokharaeian B Yazdanparast Z . BioBERT-based SNP-trait associations extraction from biomedical literature. In: ICCKE 2023 (2023). Available online at: https://ieeexplore.ieee.org/abstract/document/10326231/.

[36] Dholakia D Kalra A Misir BB Kanga U Mukerj M . HLA-spread: a natural language processing based resource for curating HLA association from pubmed abstracts. BMC Genomics. (2022). doi: 10.1101/2021.01.05.425409, PMID: 34991484 PMC8740486

